# 

**Published:** 1903-04-25

**Authors:** 


### NOTICE TO CORRESPONDENTS.

All MSS., Letters, Books for Review, and other matters intended for the Editor should be addressed The Editor, "The Hospital," The Hospital Buildings, 28 & 29 Southampton Street, Strand, W C.

The Editor cannot undertake to return rejected MS., even when accompanied by stamped directed envelope.

**Notice to Advertisers and Subscribers.**

All Advertisements, Orders for Copies of The Hospital, and Business Communications should be addressed to **THE MANAGER, (Not to the Editor)**, The Hospital, 28 & 29 Southampton Street, Strand, London, W.C.

The Cost of Subscription, post free, is as follows.—Per week, 3½d. ; per quarter, 3s. 9d.; per half-year, 7s. 6d.; per annum, 15s. Foreign and Colonial:—Per annum, 19s. 6d., payable, in all cases, in advance.

**NOTICE.—Vol. XXXIII. of "The Hospital" (October 1902 to March 1903) in handsome cloth binding, will shortly be on sale at the office of this Journal, price 7s. 6d. Cases for binding the Numbers contained in this Volume 1s. 6d. Index to Vol. XXXIII., 2½d., post free.**

**The monthly part for May will be ready on May 1. Price 6d., by post 8d.**

### The Student of Medicine.

### APPOINTMENTS.

**Charles Angus**, M.B., C.M.Aberd., appointed Medical Superintendent of the Kingseat Lunatic Asylum, Aberdeenshire. **George Ashton**, M.B., Ch.B.Vict., M.R.C.S.Eng., L.R.C.P.Lond., appointed Assistant Surgical Officer to the Manchester Royal Infirmary. **F. R. Baker**, M.R.C.S., L.R.C.P.Lond., appointed District Medical Officer of the Brighton Union. **W. L. Beaton**, M.B., Ch.B.Aberd., appointed Senior House Surgeon to the West Ham and East London Hospital, E. **F. W. Bonis**, M.D., B.Ch.Dub., appointed Assistant Medical Officer of Health to the Bury Town Council. **W. S. Campbell**, M.B., C.M Edin., appointed District Medical Officer of the Tynemouth Union. **K. D. Cooper**, L R.C.P., L.R.C.S.Edin., L.F.P.S.Glasg., appointed Resident Assistant Medical Officer of the Bradford Union Workhouse. **William Cooper**, M.D., appointed Medical Officer to Belhelvie Parish Council. **A. G. Corbyn**, M.B.Syd., appointed Medical Superintendent to the Sydney Hospital, New South Wales. **J. F. Dally, M.A.**, M.B., B.C.Cantab., L.R.C.P.Lond., M.R.C.S. Eng., appointed Assistant Resident Medical Officer to the Royal National Hospital for Consumption, Ventnor. **D. E. Darbyshire**, M.B., Ch.B.Vict., appointed Health Officer at Peppermint Grove, West Australia. **Chas. Aug. Davies**, M.D., B.S.Vict.Univ., L.S.A., appointed Admiralty Surgeon and Agent for Sick and Wounded Seamen and Marines at Ramsey, Isle of Man. **R. C. H. Forster**, M.B.Syd., appointed Government Medical Officer and Vaccinator at Narramine, New South Wales. **E. C. Foster**, M.R.C.S., L.R.C.P., appointed Junior House Surgeon to the Radcliffe Infirmary, Oxford. **G. H. Hackney**, M.R.C.S., L.R.C.P. Lond., appointed District and Workhouse Medical Officer of the Elham Union. **C. H. Hall**, M.D.Glasg., C.M., appointed Medical Officer of the Leavesden Schools of the Parish of St. Pancras. **J. A. Hutton**, M.D., B.S Durh., appointed Medical Referee under the Workmen's Compensation Act for Scarborough, Whitby, and New Malton Courts of County Court Circuit No. 16. **Francis Willan Jackson**, M.R.C.S., L.R.C.P., appointed House Physician to the West London Hospital. **R. Vandaleur Kelly**, L R.C.P. & S. Edin., C B., appointed Medical Officer to the Trial Bay Prison, New South Wales. **Wilfred Knight**, M.B., L R C.P., appointed Junior House Surgeon to the West Ham and East London Hospital. **A. A. Martin**, M.D., B.S.Lond., appointed District Medical Officer of the Eastbourne Union. **W. J. Midelton**, L.R.C.P.Lond., M.R.C.S.Eng., appointed District Medical Officer of the Christchurch Union. **L. Pern**, M.R.C.S., L.R.C.P.Lond., appointed Assistant Medical Officer of the Wandsworth and Clapham Union Infirmary. **R. H. Sankey**, M.R.C.S., L.R.C.P., appointed House Surgeon to the Radcliffe Infirmary, Oxford. **Margaret Sharpe**, L.R.C.P., L.R.C.S.Edin., appointed Assistant House Surgeon to the Middlesbrough Infirmary. **C. Simpson**, M.B., C.M.Aber., appointed District and Workhouse Medical Officer of the Towcester Union. **H. W. Spaight**, L.R.C.P., L.R.C.S.Irel., appointed District Medical Officer of the East Retford Union. **E. Taunton**, M.R.C S.Eng., L.R.C.P.Lond., appointed Assistant Medical Officer of the Whitechapel Union. **Ernest T. Keay Walker**, M.B., C.M.Glas., appointed Assistant Medical Officer to the Warneford Asylum, Oxford. **J. D. Walker**, M.B., C.M.Edin., appointed Medical Officer of Health to the Shaftesbury Town Council. **A. G. Wilkins**, M.B., Ch.B. Vict., appointed District Medical Officer of the West Ward Union. **W. Yeates**, L.R.C.P., L.R.C.S.Edin., appointed District Medical Officer of the Gateshead Union.

**"The Hospital" Institutional Library.**

"Hospitals and Asylums of the World." 4 Vols., with [a Portfolio of Plans, complete £12 12s.

"Burdett's Hospitals and Charities, 1902." 5s.

"Burdett's Official Nursing Directory, 1903." 3s. net.

"Cottage Hospitals, General, Fever, and Convalescent." 10s. 6d.

"Uniform System of Accounts for Hospitals and Public Institutions." New Edition. 4s. net.

"Hospital Expenditure: The Commissariat." 2s. 6d.

"A Legal Handbook for the Use of Hospital Authorities." 2s. 6d.

"The Cottage Hospital Case Book or Register of Patients." 10s. and 12s. 6d.

"The Furnishing and Appliances of a Cottage." 6d.

All these are published by the Scientific Press, Ltd., and may be obtained through any bookseller, or direct from the publishers, 28 and 29 Southampton Street, Strand, London, W.C.

### BOOKS THAT NURSES NEED.

**Do you want to be a Nurse?**

### THE NURSING PROFESSION: HOW AND WHERE TO TRAIN

Will give you all the information you require.

Price 2/4 post free.

**When you are a Probationer you will need—**

### THE NURSE'S DICTIONARY of MEDICAL TERMS.

By Honnor Morten. Price 2/- post free.

### NURSING: ITS THEORY and PRACTICE.

By Percy Lewis, M.D. Price 3/6 post free.

**When you are a Staff Nurse you will need—**

### A HANDBOOK FOR NURSES.

This is the most advanced text-book published for Nurses.

By J. K. Watson, M.D. Price 5/4 post free.

### SURGICAL WARD-WORK and NURSING.

By Alexander Miles, M.D. Price 3/10 post free.

**If you desire to be proficient in all branches of Nursing you will need—**

### FEVERS AND INFECTIOUS DISEASES.

By William Harding, M.D. Price 1/- post free.

### GYNÆCOLOGICAL NURSING.

By G. Hawkins-Ambler, F.R.C.S. Price 1/- post free.

### NURSING in DISEASES of the THROAT, NOSE, and EAR.

By P. M. Yearsley, F.R.C.S. Price 2/6 post free.

### OPHTHALMIC NURSING.

By Sydney Stephenson, F.R.C.S. Price 3/10 post free.

### ART OF MASSAGE.

By A. Creighton Hale. Price 6/- post free.

**All these books can be obtained at all Booksellers', and at THE SCIENTIFIC PRESS, 28 Southampton Street, Strand, W.C., who publish or sell every kind of Text-book for Nurses.**

